# Mitochondrial Phospholipid Homeostasis Is Regulated by the i-AAA Protease PaIAP and Affects Organismic Aging

**DOI:** 10.3390/cells10102775

**Published:** 2021-10-16

**Authors:** Timo Löser, Aljoscha Joppe, Andrea Hamann, Heinz D. Osiewacz

**Affiliations:** Faculty of Biosciences, Institute of Molecular Biosciences, Goethe University, 60438 Frankfurt am Main, Germany; loeser@bio.uni-frankfurt.de (T.L.); joppe@bio.uni-frankfurt.de (A.J.); a.hamann@bio.uni-frankfurt.de (A.H.)

**Keywords:** *P. anserina*, aging, mitochondria, lipid metabolism, PaIAP, PaCRD1

## Abstract

Mitochondria are ubiquitous organelles of eukaryotic organisms with a number of essential functions, including synthesis of iron-sulfur clusters, amino acids, lipids, and adenosine triphosphate (ATP). During aging of the fungal aging model *Podospora anserina*, the inner mitochondrial membrane (IMM) undergoes prominent morphological alterations, ultimately resulting in functional impairments. Since phospholipids (PLs) are key components of biological membranes, maintenance of membrane plasticity and integrity via regulation of PL biosynthesis is indispensable. Here, we report results from a lipidomic analysis of isolated mitochondria from *P. anserina* that revealed an age-related reorganization of the mitochondrial PL profile and the involvement of the i-AAA protease PaIAP in proteolytic regulation of PL metabolism. The absence of PaIAP enhances biosynthesis of characteristic mitochondrial PLs, leads to significant alterations in the acyl composition of the mitochondrial signature PL cardiolipin (CL), and induces mitophagy. These alterations presumably cause the lifespan increase of the *PaIap* deletion mutant under standard growth conditions. However, PaIAP is required at elevated temperatures and for degradation of superfluous CL synthase PaCRD1 during glycolytic growth. Overall, our study uncovers a prominent role of PaIAP in the regulation of PL homeostasis in order to adapt membrane plasticity to fluctuating environmental conditions as they occur in nature.

## 1. Introduction

Aging of biological systems is a complex and, yet, not fully understood process concerning virtually all living beings. It is primarily driven by genetic and environmental traits as well as non-predictable stochastic factors. In order to unravel the mechanistic basis of organismic aging, we use the filamentous ascomycete *Podospora anserina*, a fungal model organism closely related to *Saccharomyces cerevisiae*. Starting from a single ascospore as part of the progeny of sexual propagation, a colony (mycelium) develops, which consists of branched filamentous cells. This vegetation body grows at the hyphal tips until it reaches a strain-specific point of senescence at which growth ceases and the colony dies [1]. From more than half a century of research, it was elaborated that mitochondria play a key role in aging of *P. anserina* (reviewed in [2,3,4,5,6]). The main emphasis of this research was placed on the age-related reorganization of mitochondrial DNA (mtDNA) [7], as well as various proteins participating in essential cellular processes and pathways, ensuring a healthy population of mitochondria over time [8,9,10]. These studies provided a deep insight into molecular mechanisms of organismic aging. However, until now, we did not address the impact of phospholipids (PLs) on mitochondria-related aging processes, even though they are key components of the membrane layers surrounding these organelles. Numerous studies have demonstrated that the distribution of PLs, such as cardiolipin (CL), phosphatidylcholine (PC), and phosphatidylethanolamine (PE), has major impacts on overall mitochondrial function and quality [11,12,13]. Due to their different structural and biochemical properties, these PLs are able to interact physically with various mitochondrial proteins, involved in respiration [14], metabolite trafficking [15], and membrane dynamics [16]. Therefore, it is not surprising that more recently mitochondrial phospholipids have come into focus as important regulators of aging and age-related diseases [17,18,19].

In the current study, for the first time, we unraveled the mitochondrial PL profile of *P. anserina*, thereby establishing a link between mitochondrial PL distribution and aging. Moreover, we investigated a strain in which PaIAP is ablated, a mitochondrial protease homologous to yeast YME1. We report that this protease is involved in the control of PL homeostasis and is essential for the ability of the fungus to adapt to natural fluctuations of environmental conditions.

## 2. Materials and Methods

### 2.1. P. anserina Strains and Cultivation

The *P. anserina* wild-type strain “*s*” [1], *∆PaIap* [10], and newly generated *∆PaCrd1, ΔPaTaz1, PaCrd1OEx1, PaSod3^H26L^::mCherry*, and *∆PaIap/PaSod3^H26L^::mCherry* strains were used in this study. If not otherwise stated, strains were cultivated on solid M2 medium at 27 °C under constant light conditions [20]. Monokaryotic ascospores were germinated on BMM containing 60 mM ammonium acetate for 2 days at 27 °C in the dark and subsequently used for all performed experiments. For lifespan analysis, the strains were inoculated in race tubes containing M2 medium. For mitochondria isolation, strains were either grown on M2 agar plates covered with cellophane foil as previously described in [21], or on M2 with 1% (*w*/*v*) glycerol (M2G) as the primary carbon source. After 3 days, grown mycelium was transferred to liquid CM medium with either 1% (*w*/*v*) glucose or 1% (*w*/*v*) glycerol (CMG) and incubated at 27 °C with shaking for 2 days. For isolation of total protein extract, strains were grown on M2 solid (3 days) and CM liquid medium (2 days, shaking) as described in [21]. For RNA isolation, all strains were grown on either M2 or M2G agar plates covered with cellophane foil. To investigate iron stress tolerance, germinated strains were grown for 3 days on M2 solid medium supplemented with either 0, 50, 200, 300 or 500 µM FeCl_3_ (Carl Roth, Karlsruhe, Germany, P742.1). For heat stress analysis, strains were grown on M2 medium for 3 days. Subsequently, same-sized pieces of mycelium were transferred to liquid CM medium and inoculated for 1 day at 27 °C with shaking.

### 2.2. Lifespan Analysis

Strains used for lifespan experiments were inoculated in race tubes containing M2 medium with either glucose or glycerol as carbon source and grown at 27 °C under constant light until senescence. Lifespan is defined as surviving cultures (%) per day.

### 2.3. Cloning Procedure and Generation of P. anserina Mutants

All strains were generated in the genetic background of the wild-type strain “*s*” [1]. Construction of *∆PaCrd1* and *∆PaTaz1* was performed using the *ΔPaKu70* strain in which the frequency of homologous recombination is increased [22]. To this end, 1 kb flanking regions of *PaCrd1* (corresponding to UniProt B2AX19) or *PaTaz1* (UniProt B2AWG5) were amplified with oligonucleotides Crd1KO5 or Taz1KO5 (AAGGTACCAGGTGACTTGGGCATTTG, respectively, AAGGTACCGGCTCTAGACATAGCCAG, KpnI site underlined, Biomers, Ulm, Germany) and Crd1KO6 or Taz1KO6 (GCCGATCGATGGTGAATGTTCGAAACAG, respectively, GGATCGATGCGGAGTTCTTGGGATTG, ClaI site underlined, Biomers, Ulm, Germany), for 5′-flank, and Crd1KO7 or Taz1KO7 (AACTGCAGGGAGAAGATGAGGGTGAG, respectively, AGCTGCAGGGATTAGGAGACTTGAGG, PstI site underlined, Biomers, Ulm, Germany) and Crd1KO8 or Taz1KO8 (ACGGATCCTACTCCCTCCTCGAAAGC, respectively, AAGGATCCAGGTGTTGTCGGGTATGC, BamHI site underlined, Biomers, Ulm, Germany) for the 3′-flanking region. First, the 3′-flank was cloned into plasmid pKO7 [23] digested with PstI/BamHI (Thermo Scientific, Waltham, MA, USA, ER0611, ER0051) producing plasmid pCrd1KO3, respectively pTaz1KO3. Subsequently the 5′-flank of *PaCrd1* or *PaTaz1* was cloned into the KpnI and ClaI (Thermo Scientific, Waltham, MA, USA, ER0521, ER0141) sites of pCrd1KO3 or pTaz1KO3 leading to the plasmids pCrd1KO4 and pTaz1KO4. A total of 10 µg of these plasmids was transformed into protoplasts of *∆PaKu70* spheroplasts as previously described [24], selection was performed on hygromycin B (Calbiochem, San-Diego, CA, USA, 400051) containing medium. Correct replacement of the *PaCrd1* or the *PaTaz1* gene (Figure S1) was monitored with a Southern blot analysis. Since a suitable antibody for PaCRD1 was available, ablation of the protein was additionally demonstrated by western blot analysis.

*PaCrd1* overexpression was achieved with plasmid pCrd1Ex1, which contains the *PaCrd1* open reading frame (ORF) and terminator region under control of the constitutive minimal promoter of the metallothionein gene *PaMt1* [25]. Therefore, the *PaCrd1* ORF and terminator were amplified with oligonucleotides Crd1Ex1 (AAGGATCCATGTCCTCCCCCCCGCTC, BamHI site underlined, Biomers, Ulm, Germany) and Crd1Ex2 (GGGAAGCTTGGGAGCAAGGCGCTGGATTTG, HindIII site underlined, Biomers, Ulm, Germany), digested with BamHI, HindIII (Thermo Scientific, Waltham, MA, USA, ER0051, ER0501) and cloned into plasmid pExMthph [26], conferring hygromycin B resistance. Subsequently, 10 µg of the resulting plasmid pCrd1Ex1 were transformed into spheroplasts of wild-type strain “*s*” according to standard protocols [21]. One of the transformants with a single ectopic integration of the plasmid (identified by Southern blot analysis) was further investigated and demonstrated to contain elevated PaCRD1 levels.

To construct the mitophagy reporter *PaSod3^H26L^::mCherry*, the *Gfp* gene in plasmid pSM4 [27] was removed with EcoRI/BamHI (Thermo Scientific, Waltham, MA, USA, ER0271, ER0051) and replaced by the *mCherry* gene, amplified from plasmid pmCherry (Clontech, Mountain View, CA, USA, 6325229) with oligonucleotides mCherry-1 (CGGCGAATTCCATGGTGAGCAAGGGCGAG, EcoRI site underlined, Biomers, Ulm, Germany) and mCherry-2 (GCGGATCCCTACTTGTACAGCTCGTC, BamHI site underlined, Biomers, Ulm, Germany). The resulting plasmid pLS1 contains the *mCherry* gene fused at the 3′-end with the *Aspergillus nidulans* trpC terminator. Subsequently, a mutated, catalytically inactive version of *PaSod3* encoding mitochondrial superoxide dismutase (promoter and ORF) was amplified from plasmid pPaSod3^H26L^-Gfp [28] with oligonucleotides Sod3mCherry1 (AGCATCGATTCTGAGGAACTCCTCACC, ClaI site underlined, Biomers, Ulm, Germany) and Sod3mCherry2 (CGCCGAATTCAAGTCATATCTCTTGGCAAC, EcoRI site underlined, Biomers, Ulm, Germany). The corresponding PCR product was digested with ClaI/EcoRI (Thermo Scientific, Waltham, MA, USA, ER0141, ER0271) and cloned into pLS1 ClaI/EcoRI. The resulting plasmid, pSod3^H26L^-mCherry encodes catalytically inactive mitochondrial PaSOD3^H26L^ C-terminally fused to mCHERRY. 10 µg of pSod3^H26L^-mCherry were transformed into spheroplasts of wild-type strain “*s*” according to standard protocols [21]. One of the transformants with single integration of the plasmid (identified by Southern blot analysis) was used as a mitophagy reporter strain.

The *∆PaIap/PaSod3^H26L^::mCherry* double mutant was generated after crossing the two single mutant strains and subsequent selection of recombinant progeny containing both mutations.

### 2.4. Southern Blot Analysis

DNA isolation from *P. anserina* was performed according to the protocol of Lecelier and Silar [29]. DNA restriction, agarose gel electrophoresis and Southern blotting were performed by standard protocols [30]. DNA hybridization and detection were executed as described in the protocol for digoxigenin-labeled hybridization probes (DIG DNA Labeling Mix, Sigma-Aldrich, St. Louis, MO, USA, 11175033910). The *PaCrd1*-specific fragment was generated by PCR mediated amplification from pCrd1Ex1 with the oligonucleotides Crd1-1 (GCGGACAAGATGTTGATG, Biomers, Ulm, Germany) and Crd1-2 (CGCATCCTTGCTGAACAC, Biomers, Ulm, Germany). The *PaTaz1*-specific fragment was obtained by PCR with oligonucleotides Taz1-1 (TCAGTTCAGGGAGCTGTTG, Biomers, Ulm, Germany) and Taz1-2 (GTGAAGAAGGAGGAGAAG, Biomers, Ulm, Germany). The hygromycin resistance gene *hph* was detected with a NcoI/ClaI-digested (Thermo Scientific, Waltham, MA, USA, ER0571, ER0141) fragment from the pKO7 plasmid. The *PaIap* gene was detected with a NcoI-digested fragment encompassing a part of the open reading frame of *PaIap*. A specific *mCherry* fragment was amplified from the pLS1 plasmid with the oligonucleotides mCherry-P (ATGGTGAGCAAGGGCGAGGA, Biomers, Ulm, Germany) and mCherryR-ClaI (GCCCATCGATCTTGTACAGCTCGTCCATGC, Biomers, Ulm, Germany).

### 2.5. Mitochondria Isolation

Isolation and purification of mitochondria from *P. anserina* were performed as previously described in [21]. Briefly, grown mycelia were disrupted in isotonic mitochondria isolation buffer in presence of 0.2% (*w*/*v*) bovine serum albumin (BSA) (Sigma-Aldrich, St. Louis, MO, USA, A6003) and filtered through nettle cloth. The filtrate was sedimented by centrifugation (12,000× *g*), resuspended in isolation buffer lacking BSA, and loaded onto a sucrose gradient (20–50% sucrose in water (*w*/*v*)). Separation of intact mitochondria from disrupted mitochondrial fractions and vacuoles was performed by ultracentrifugation (100,000× *g*). Mitochondria were collected, sedimented (15,000× *g*) and resuspended in isolation buffer. Isolates were stored at −80 °C.

### 2.6. Cycloheximide Assay

In order to monitor protein degradation after disrupting cytoplasmatic protein biosynthesis, 200 µg cycloheximide (in ethanol p.a.) (Sigma-Aldrich, St. Louis, MO, USA, 66-81-9) was added to the growing strains in CM medium 24 h prior to mitochondria isolation. Growth conditions and isolation of mitochondria was performed as described in *“Mitochondria Isolation”*.

### 2.7. Total Protein Extraction

The total protein of *P. anserina* strains was isolated as described in [31]. Briefly, grown mycelia were disrupted in protein isolation buffer containing 5 mM dithiothreitol (DTT) (Carl Roth, Karlsruhe, Germany, 6908.4) and subsequently sedimented at 9300× *g*. The supernatant containing total protein was collected and stored at −20 °C.

### 2.8. Lipidomic Analysis

Mass spectrometry-based lipid analysis was performed by Lipotype GmbH (Dresden, Germany) as described in [32,33]. Lipids were extracted using a two-step chloroform/methanol procedure [32]. Samples were spiked with internal lipid standard mixture containing: CDP-diacylglycerol 17:0/18:1 (CDP-DAG), ceramide 18:1;2/17:0 (Cer), cardiolipin 14:0/14:0/14:0/14:0 or 15:0/15:0/15:0/16:1 (CL), diacylglycerol 17:0/17:0 (DAG), lyso-phosphatidic acid 17:0 (LPA), lyso-phosphatidylcholine 12:0 (LPC), lyso-phosphatidylethanolamine 17:1 (LPE), lyso-phosphatidylinositol 17:1 (LPI), lyso-phosphatidylserine 17:1 (LPS), phosphatidic acid 17:0/14:1 (PA), phosphatidylcholine 17:0/14:1 (PC), phosphatidylethanolamine 17:0/14:1 (PE), phosphatidylglycerol 17:0/14:1 (PG), phosphatidylinositol 17:0/14:1 (PI), phosphatidylserine 17:0/14:1 (PS), ergosterol ester 13:0 (EE), triacylglycerol 17:0/17:0/17:0 (TAG), inositol phosphorylceramide 44:0;2 (IPC), mannosyl-inositol-phosphorylceramide 44:0;2 (MIPC), and mannosyl-di-(inositol-phosphoryl)ceramide 44:0;2 (M(IP)_2_C). After extraction, the organic phase was transferred to an infusion plate and dried in a speed vacuum concentrator. The first step, dry extract was re-suspended in 7.5 mM ammonium acetate in chloroform/methanol/propanol (1/2/4; *v*/*v*/*v*), and the second step, dry extract in 33% ethanol solution of methylamine in chloroform/methanol (0.003/5/1; *v*/*v*/*v*). All liquid handling steps were performed using Hamilton Robotics STARlet robotic platform (Reno, NV, USA) with the Anti Droplet Control feature for organic solvents pipetting.

Samples were analyzed by direct infusion on a QExactive mass spectrometer (Thermo Scientific, Waltham, MA, USA) equipped with a TriVersa NanoMate ion source (Advion Biosciences, Ithaca, NY, USA). Samples were analyzed in both positive and negative ion modes with a resolution of R_m/z=200_ = 280,000 for MS and R_m/z=200_ = 17,500 for MSMS experiments, in a single acquisition. MSMS was triggered by an inclusion list encompassing corresponding MS mass ranges scanned in 1 Da increments [34]. Both MS and MS/MS data were combined to monitor EE, DAG, and TAG ions as ammonium adducts; PC as an acetate adduct; and CL, PA, PE, PG, PI, and PS as deprotonated anions. MS was only used to monitor LPA, LPE, LPI, LPS, IPC, MIPC, M(IP)_2_C as deprotonated anions, and Cer and LPC as acetate adducts.

Data were analyzed with in-house developed lipid identification software based on LipidXplorer [35,36]. Data post-processing and normalization were performed using an in-house developed data management system. Only lipid identifications with a signal-to-noise ratio > 5, and a signal intensity 5-fold higher than in corresponding blank samples were considered for further data analysis.

### 2.9. Thin-Layer-Chromatography (TLC)

Phospholipid (PL) extraction from isolated mitochondria was performed as described by Bligh and Dyer [37]. Therefore, 200 µg mitochondria were treated with 750 µL of a chloroform/methanol mixture (1/2; *v*/*v*) and incubated on ice for 30 min. Subsequently, 250 µL of each, chloroform and water were added and the sample was mixed thoroughly. Organic phase was extracted twice after phase separation by centrifugation (90× *g*, 5 min, room temperature). Remaining organic solvent was evaporated and the dried lipids were diluted in 10 µL chloroform/methanol (1/1; *v*/*v*). Lipid samples were used immediately.

Separation of extracted PLs was performed on TLC Silica gel 60 F_254_ plates (10 cm × 20 cm, Supelco, Bellefonte, PA, USA, 1057290001). Plates were washed in methanol/chloroform (1/1; *v*/*v*) and dried under a fume hood. The dried plates were impregnated with 1.8% (*w*/*v*) boric acid (Merck, Darmstadt, Germany, 1.00165.1000), dried and activated in an oven at 100 °C for 15 min. Total 10 µL of extracted lipids as well as lipid markers cardiolipin (18:1, Avanti^®^, Alabaster, AL, USA, 710335C) phosphatidylcholine (18:1, Avanti^®^, Alabaster, AL, USA, 850375P), phosphatidylethanolamine (18:1, Sigma-Aldrich, St. Louis, MO, USA, 42490), phosphatidylglycerol (18:1, Avanti^®^, Alabaster, AL, USA, 840475C), and phosphatidylserine (18:1, Avanti^®^, Alabaster, AL, USA, 840035C), each 1 mg/mL in chloroform/methanol (1/1; *v*/*v*), were spotted on the lower end of the plate (1 cm from bottom). Subsequently, the plate was developed in a prepared solvent system containing chloroform/methanol/acetic acid/water (7.5/1.5/0.75/0.35; *v*/*v*/*v*/*v*) until the mobile phase reached the end of the plate (approximately 2 h). The TLC plate was dried under a fume hood, subsequently treated with 10% (*v*/*v*) sulfuric acid in methanol, and baked at 150 °C for 10–15 min until lipid spots became visible.

### 2.10. Western Blot Analysis

Western blot analysis was performed as described in [31] with either 50 µg mitochondria or 100 µg total protein extract. For this study, the following primary antibodies were used: anti-PaCRD1 (rabbit, dilution 1:5000, peptide: [H]-SKKEKEVVVEEEEGKKKEL-[OH], Davids Biotechnologie GmbH, Regensburg, Germany), anti-PaIAP (rabbit, dilution 1:2500, peptide: [H]-KAENQKARFSDVHGC-[OH], Sigma-Genosys, Haverhill, United Kingdom), anti-mCHERRY (mouse, dilution 1:3000, Sigma-Aldrich, St. Louis, MO, USA, SAB2702286), anti-PaPSD1 (rabbit, dilution 1:1000, peptide: [H]-DTEADRAAEEAAQRGEPYLG-[OH], Davids Biotechnologie GmbH, Regensburg, Germany), anti-PaGEP4 (rabbit, dilution 1:1000, peptide: [H]-DKAFAGQREKVDIKAVVLDKDD-[OH], Davids Biotechnologie GmbH, Regensburg, Germany), anti-PaMDM35 (rabbit, dilution 1:1000, peptide: [H]-KERGIDKLLDEAREDFKENDA-[OH], Davids Biotechnologie GmbH, Regensburg, Germany), anti-PaPORIN (rabbit, dilution 1:5000, full length protein, Sigma NEP, Gardner, Massachusetts, USA), anti-PaACO2 (rabbit, dilution 1:10,000, peptide: [H]-KRTEIGDFARSYAKELRQDDG-[OH], Davids Biotechnologie GmbH, Regensburg, Germany), anti-PaRIESKE (rabbit, dilution 1:1000, peptide: [H]-ASLRDPEADSDRVQKPE-[OH], Davids Biotechnologie GmbH, Regensburg, Germany), IR Dye^®^ 680RD anti-rabbit (goat, dilution 1:15,000, LI-COR Biosciences, Bad Homburg, Germany, 926-68071), and IRDye^®^ 680RD anti-mouse (goat, dilution 1:15,000, LI-COR Biosciences, Bad Homburg, Germany, 926-68070) were used as conjugated secondary antibodies.

### 2.11. Blue-Native Polyacrylamide Gel Electrophoresis (BN-PAGE)

BN-PAGE of isolated mitochondria was performed according to [38]. Therefore, 100 µg mitochondria were solubilized using digitonin (Sigma-Aldrich, St. Louis, MO, USA, D141) in a ratio of digitonin/mitochondria = 4/1 (*w*/*w*). Before Coomassie-staining, separated protein complexes were fixated for 30 min in fixating solution (50% (*v*/*v*) methanol, 10% (*v*/*v*) acetic acid and 100 mM ammonium acetate in water).

### 2.12. Quantitative Real-Time PCR (qPCR)

RNA isolation and subsequent cDNA synthesis was performed as described in [39]. In order to determine transcript levels of *PaCrd1* the oligonucleotides Crd1-1 (GCGGACAAGATGTTGATG, Biomers, Ulm, Germany) and Crd1-2 (CGCATCCTTGCTGAACAC, Biomers, Ulm, Germany) were used. Expression levels were normalized to transcript level of *PaPorin*, which was determined by usage of oligonucleotides Porin-RT-for (TCTCCTCCGGCAGCCTTG, Biomers, Ulm, Germany) and Porin-RT-rev (CGGAGGCGGACTTGTGAC, Biomers, Ulm, Germany). For both genes, PCR efficiency was determined as described in [40].

### 2.13. Stress Sensitivity Assays

For stress assays, freshly germinated monokaryotic wild-type and *ΔPaIap* strains were used. Iron tolerance was investigated by growing each strain on solid M2 medium supplemented with 0, 50, 200, 300, or 500 µM FeCl_3_. After 3 days, growth rate of each strain was determined in cm/d. In order to investigate heat stress tolerance, equally sized pieces of 6-day-old wild-type and *ΔPaIap* mycelium were incubated in liquid CM medium at 27 or 42 °C for 5 h and subsequently shifted to M2 solid medium for inoculation at 27 °C. After 3 days, growth rate (cm/d) was determined for each individual.

### 2.14. Determination of ROS Release

In order to determine the release of superoxide anion free radical as well as hydrogen peroxide, histochemical NBT, respectively, DAB staining was performed according to [41].

### 2.15. Statistical Analysis

Each statistical analysis was performed by a two-tailed Student’s t-test. *p < 0.05, **p < 0.01 ***, *p* < 0.001. Error bars in all figures resemble standard deviations (SD).

## 3. Results and Discussion

### 3.1. Unravelling Age-Dependent Alterations in the Mitochondrial Lipid Composition of P. anserina

**Figure 1:**
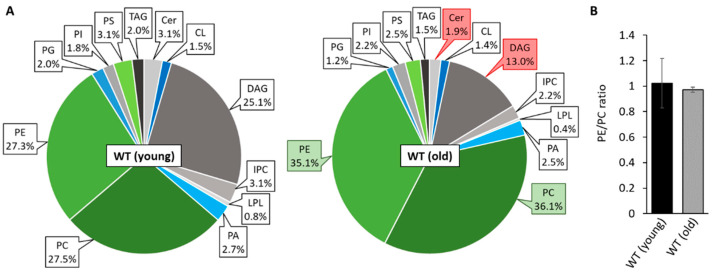
Mitochondrial PL composition changes during aging. (**A**): PL composition of isolated mitochondria of 7-day-old (young) and 21-day-old (old) wild-type (WT) cultures grown under glycolytic conditions. Data represent relative proportion of total lipids. Significantly (*p* < 0.05) increased or reduced PLs are marked with green and red boxes, respectively. Exact values (mean ± SD) can be found in supplementary material (Table S1). CL: cardiolipin, Cer: ceramide; DAG: diacylglycerol; IPC: inositol phosphorylceramide; LPL: lyso-phospholipids; PA: phosphatidic acid; PC: phosphatidylcholine; PE: phosphatidylethanolamine; PG: phosphatidylglycerol; PI: phosphatidylinositol; PS: phosphatidylserine; TAG: triacylglycerol. (**B**): PE/PC ratio of 7 and 21-day-old wild type. Data represent mean ± SD (young: n = 11; old: n = 5).

Previous studies revealed substantial age-associated changes of mitochondrial morphotypes in *P. anserina* [42] and massive remodeling of the IMM leading to a shift from lamellar cristae to a vesiculated ultrastructure [43]. In order to elucidate the impact of mitochondrial lipids on aging in *P. anserina*, we set out to analyze the membrane composition of the wild type in different age stages. Therefore, we isolated and purified mitochondria from young (7-day-old) and old (21-day-old) wild-type “*s*” cultures of *P. anserina* for shotgun lipidomic analysis performed by Lipotype (Dresden, Germany). As depicted in Figure 1, the most abundant mitochondrial membrane lipids are phosphatidylcholine (PC), phosphatidylethanolamine (PE) and diacylglycerol (DAG). In young individuals, levels of PE and PC with a proportion of 25–30% and a PE/PC ratio of approximately 1.0 (Figure 1A,B) are similar to those in isolated mitochondria of other fungi including *S. cerevisiae* [44] and *Neurospora crassa* [45]. The abundance of DAG was found to be rather high with 25% of the analyzed PLs, possibly indicating a co-purification of mitochondria-attached ER or lipid droplets. Nevertheless, the amount of DAG is comparable to previous observations in highly purified yeast mitochondria [46], which is why we expected the quality of the isolated mitochondrial fractions to be sufficient for further experiments. Surprisingly, we detected only low amounts of the mitochondrial signature PL cardiolipin (1.5%), which, in other species like yeast, mammals or plants, represents 10–15% of mitochondrial PLs [47]. During aging, PE and PC levels increase significantly in *P. anserina* and represent almost ¾ of total lipids in 21-day-old cultures, while ceramide (Cer) as well as the precursor lipid DAG decrease in abundance (Figure 1B). In contrast, CL levels are preserved during aging. These data raise the question in how far altered levels of mitochondrial PLs affect fungal development and whether manipulation of the mitochondrial lipid profile may affect the process of aging in *P. anserina*.

### 3.2. Manipulating Components of PL Biosynthesis Modulates Mitochondrial Lipid Composition and Affects Lifespan

**Figure 2:**
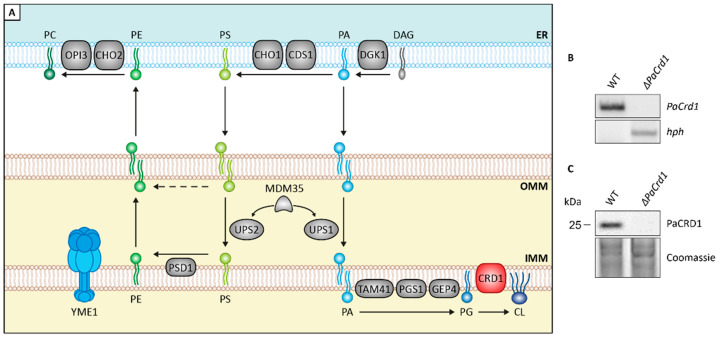
Genetic modulation of PL homeostasis. (**A**): Scheme depicting major components of PL biosynthesis pathways in yeast. ER: endoplasmatic reticulum; OMM: outer mitochondrial membrane; IMM: inner mitochondrial membrane; CDS1: PA cytidylyltransferase; CHO1: PS synthase; CHO2: PE methyltransferase; CRD1: CL synthase; DGK1: DAG kinase; GEP4: phosphatidylglycerophosphatase; OPI3: PL methyltransferase; PGS1: phosphatidylglycerophosphate synthase; PSD1: PS decarboxylase proenzyme 1; TAM41: PA cytidylyltransferase; UPS1: unprocessed MGM1 protein 1; UPS2: unprocessed MGM1 protein 2; YME1: yeast mitochondrial escape protein 1. For other abbreviations, see caption of Figure 1. (**B**): Southern blot analysis with HindIII-digested DNA of *P. anserina* wild-type (WT) and *ΔPaCrd1* strains demonstrating replacement of the *PaCrd1* ORF with a hygromycin resistance gene (*hph*) in the deletion mutant. (**C**): Western blot analysis of PaCRD1 levels in isolated mitochondria of WT and *ΔPaCrd1*. Coomassie was used as loading control.

Next, we set out to investigate the impact of altered mitochondrial lipid metabolism on aging of *P. anserina* by genetically modifying components of the CDP-DAG pathway. This pathway, which is highly conserved in eukaryotes, is responsible for *de novo* synthesis of the most abundant PLs in mitochondria, PC, PE, and CL (Figure 2A). It relies on a complex network of lipid trafficking between the endoplasmatic reticulum (ER) and mitochondria as well as various PL synthesizing and remodeling steps. In yeast, the initial step of this pathway is the transport of the ER-located precursor PLs phosphatidic acid (PA), which is synthesized from DAG by CTP-dependent diacylglycerol kinase DKG1 and phosphatidylserine (PS), derived from PA catalyzed by PA cytidylyltransferase CDS1 and PS synthase CHO1, to the outer mitochondrial membrane (OMM) via membrane contact sites [48]. Subsequently, two lipid transfer proteins, unprocessed MGM1 protein 1 and 2 (UPS1 and UPS2), mediate the transport of PA and PS across the intermembrane space (IMS) [49,50]. For both proteins, a heterodimeric interaction with mitochondrial distribution and morphology protein 35 (MDM35) ensures protein stability [51] and allows the carriage of these precursors to the IMM. Being released in the IMM, PA undergoes a cascade of enzyme-dependent synthesizing steps by PA cytidylyltransferase TAM41, phosphatidylglycerophosphate synthase PGS1 and phosphatidylglycerophosphatase GEP4, ultimately resulting in the conversion of phosphatidylglycerol (PG) to CL by CL synthase CRD1. PS, on the other hand, is decarboxylated to PE by PS decarboxylase proenzyme 1 (PSD1). In contrast to CRD1, the catalytic center of membrane bound PSD1 reaches into the IMS [52,53]. Therefore, PE can also be generated in the OMM independently of PS transport at membrane contact sites [54]. Newly generated PE can either be accumulated in the mitochondrion or exported to the ER where it is trimethylated to PC by PE specific methyltransferase CHO2 and PL methyltransferase OPI3 [55]. End products of this synthesis machinery can be modulated by mitochondrial transacylases, such as tafazzin (TAZ1) [56].

In the current study, we included two *P. anserina* mutants, which are both likely to regulate mitochondrial PL biosynthesis. One of these mutants is a deletion mutant of *PaCrd1*, being unable to catalyze the PaCRD1 mediated conversion of PG to CL (Figure 2B,C). The second strain is a previously generated *ΔPaIap* mutant [10], lacking the i-AAA protease PaIAP (yeast YME1 homologue). In yeast, YME1 is known to be responsible for degradation of several proteins involved in PL metabolism [51].

**Figure 3:**
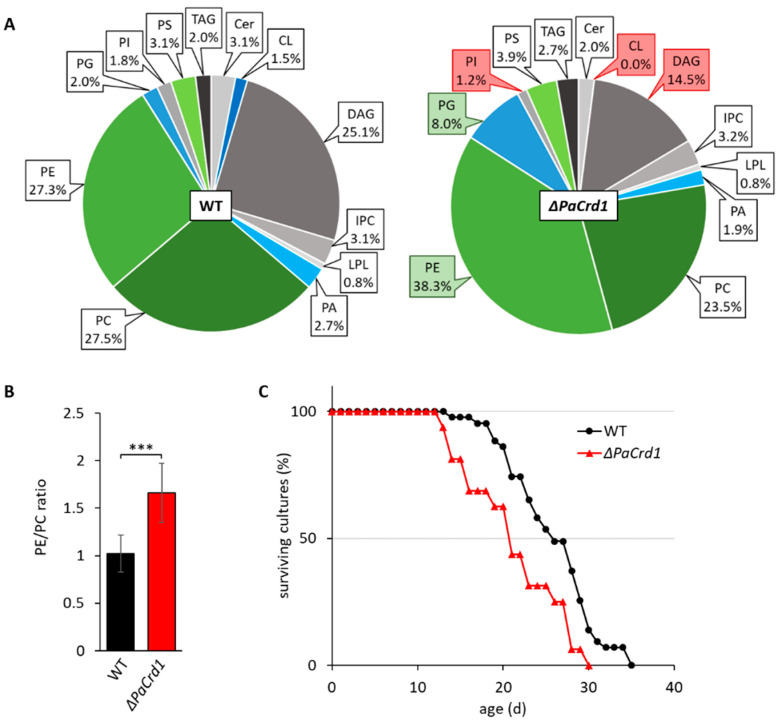
Deletion of *PaCrd1* dramatically affects PL metabolism and lifespan. (**A**): PL composition of isolated mitochondria of 7-day-old wild-type (WT) and *ΔPaCrd1* strains grown under glycolytic conditions. Data represent relative proportion of total lipids. Significantly (*p* < 0.05) increased or reduced PLs are marked with green and red boxes, respectively. Exact values can be found in supplementary material (Table S1). For abbreviations, see caption of Figure 1. (**B**): PE/PC ratio of wild type and *ΔPaCrd1*. Data represent mean ± SD (n = 5). *** *p* < 0.001. (**C**): Lifespan analysis of wild type (n = 43) and *ΔPaCrd1* (n = 16) grown on M2 medium at 27 °C.

The low amount of CL in wild-type strains led us to question whether CL plays an important role in mitochondrial function of *P. anserina*, especially since it represents an essential factor for many mitochondrial lipid-protein interactions in other organisms. By analyzing the *PaCrd1* deletion strain, we directly addressed effects of total loss of CL. As expected, the ablation of PaCRD1 results in the absence of mitochondrial CL accompanied by an accumulation of PG up to 8% (Figure 3A). Interestingly, the elevated PG levels seem to not only result from impaired conversion to CL, but also from an increased level of PG synthesizing PaGEP4 (Figure S2). PaGEP4 is presumably upregulated to compensate the loss of CL. Additionally, we detected slightly reduced PI levels as well as a vast enrichment of PE up to 38% at the expense of the precursor lipid DAG. In yeast, strongly elevated PG levels were shown to cause perturbations in mitochondrial morphology and function [57,58]. Further, the high abundance of PE leads to a dramatic shift of the PE/PC ratio from 1.0 to 1.7 (Figure 3B), which has major impact on membrane properties, since PC is a cylindrical bilayer-forming PL while cone-shaped PE induces negative membrane curvature [59] and increases membrane stiffening [60]. In mammalian cells alterations of the PE/PC ratio was shown to drastically affect different cellular processes, including mitochondrial dynamics and respiration [61]. Consistent with studies in yeast cells, it is also most likely that the increase of PE leads to cellular stress and a protein independent activation of the unfolded protein response (UPR) [44,62,63,64]. Accordingly, we observed that the deletion of *PaCrd1* causes impaired growth and a shortened mean lifespan by 18% (Figure 3C), presumably due to the absence of CL and elevated PE and PG levels. This implies that despite the low amount of 1.5% of CL in wild-type strains, mitochondrial CL synthesis is important for proper growth and development. Moreover, these findings indicate that genetic manipulation of mitochondrial lipid metabolism is able to impact aging in *P. anserina* as it affects lifespan under standard conditions.

PL homeostasis cannot only be affected directly by genetically modifying the level of PL synthesizing enzymes but also indirectly by manipulation of components that regulate the level of those proteins. In yeast cells, such a regulator is the i-AAA protease YME1, which is involved in the turnover of several components of the CDP-DAG pathway, such as the PL transport proteins UPS1 and UPS2 [51] as well as PSD1 [65]. In *P. anserina*, ablation of the YME1 homologue PaIAP was previously shown to cause an extended lifespan of approximately 180% compared to wild-type strains [10]. Even though factors like growth, fertility and mycelial pigmentation were wild-type-like and mitochondrial structure showed no abnormalities [66], the mutant demonstrates significant changes at a deeper mitochondrial level. For instance, in *ΔPaIap*, assembly of respiratory chain supercomplexes (RCS) is vastly increased [10]. Formation of RCS is known to improve electron flow between respiratory complexes therefore counteracting generation of reactive oxygen species (ROS) at complexes I and III [67,68]. Interestingly, we observed no visible differences of the superoxide anion free radical or of hydrogen peroxide release between juvenile wild-type and *ΔPaIap* strains (Figure S3). These data are in accordance with earlier data which did not reveal significant differences of ROS-induced protein carbonylation in both strains [65].

**Figure 4:**
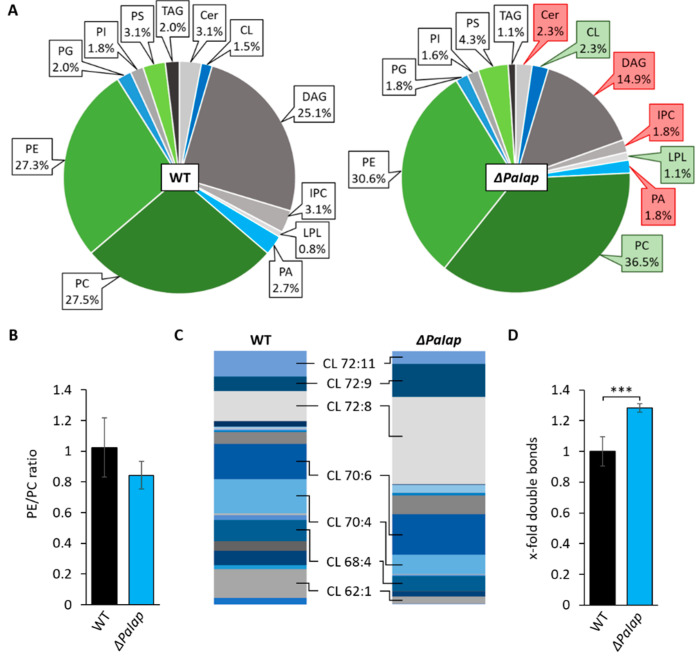
Deletion of *PaIap* alters mitochondrial lipid profile. (**A**): Comparative PL composition of isolated mitochondria of 7-day-old wild-type (WT) strains and *ΔPaIap* mutants grown under glycolytic conditions. Data represent relative proportion of total lipids. Significantly (*p* < 0.05) increased or reduced PLs are marked with green and red boxes, respectively. Exact values can be found in supplementary material (Table S1). For abbreviations, see caption of Figure 1. (**B**): PE/PC ratio of WT and *ΔPaIap*. Data represent mean ± SD (n = 5). (**C**): Graphical illustration of relative proportions of different CL species in wild type (WT) and *ΔPaIap* according to total length of all 4 acyl chains (58–72) and total degree of unsaturation (1–11), e.g., CL 72:8. The displayed species represent a selection of the most abundant CL variants in both strains (proportion > 8%). (**D**): Comparative analysis of total double bonds across all CL species. Total amount of double bonds in CL species was related to wild type (set to 1). Data represent mean ± SD (n = 5). *** *p* < 0.001. Exact values can be found in supplementary material (Table S2).

Additionally, it was shown that deletion of *PaIap* leads to an increased mtDNA stabilization during aging [66]. In *S. cerevisiae*, improved RCS assembly as well as mtDNA stabilization was already correlated with interactions mediated by the anionic membrane lipid CL [69]. We therefore speculated that loss of PaIAP may affect CL metabolism. As CL was recently shown to be crucial for maturation of frataxin, a conserved mitochondrial protein being necessary for iron-sulfur biogenesis [70], we also tested the iron sensitivity of the mutant. We found that *ΔPaIap* demonstrates a slightly improved tolerance to free iron cations (Figure S4). Taken together, these observations strongly suggest that loss of PaIAP leads to increased CL metabolism. Consequently, we investigated the mitochondrial PL profile of this mutant strain and detected a significant increase of CL, PC, as well as slightly, but not significantly, elevated levels of PE (Figure 4A), all of which represent PL end products of the CDP-DAG pathway. Lyso-PLs (LPLs) were also moderately increased. Simultaneously, we observed a reduction of precursor lipids like PA and DAG, as well as Cer and inositol-phosphorylceramide (IPC).

In a more specific analysis, we also discriminated the different species of CL concerning length and degree of saturation of attached acyl groups. The data revealed an altered distribution of different acyl species compared to the wild type (Figure 4C). We found that in wild-type strains the proportions of all present CL species were rather equal, whereas the *ΔPaIap* mutant showed a significant increase in tetra-linoleoyl containing CL (CL 72:8). This particular species is formed by CL remodeling enzyme tafazzin (PaTAZ1) (Table S2C) and was previously demonstrated as the predominant CL variant in cardiac mitochondria of mammals [71], which are known for exceptionally high respiratory activity. Further, studies also revealed that elevated CL 72:8 levels improved mitochondrial function in rat models with severe heart failures [72,73]. The elevated amounts of CL 72:8 consequently led to a nearly 30% increase of double bonds across all CL molecules compared to wild-type CL (Figure 4D). Higher degrees of unsaturation are shown to contribute to enhanced membrane fluidity (as reviewed in [74]). These findings raised the question whether the increase in specific mitochondrial lipids as well as the altered composition of CL species trigger the phenotype of this *P. anserina* deletion mutant and, in particular, what evolutionary benefit this protease has to offer at all. Particularly since the *PaIap* deletion strain is characterized by a strongly extended lifespan, despite the absence of a putatively essential component of mitochondrial quality control.

### 3.3. Absence of PaIAP Promotes Cardiolipin Synthase Activity by Loss of Proteolytic Turnover

**Figure 5:**
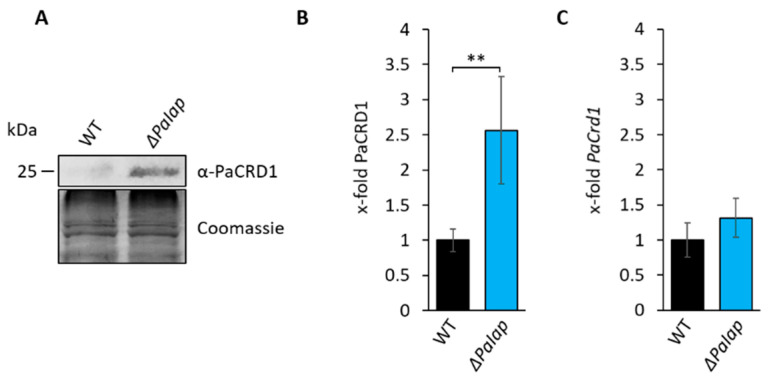
Deletion of *PaIap* prevents proteolytic turnover of PaCRD1. (**A**): Western blot analysis of isolated mitochondria of 7-day-old wild type (WT) and *ΔPaIap*. Coomassie staining was used as loading control. (**B**): Quantification of PaCRD1 in (**A**). PaCRD1 levels were determined and related to wild type (set to 1). Data represent mean ± SD (n = 5). ** *p* < 0.01. (**C**): Transcript analysis of 5-day-old wild type and *ΔPaIap* grown on M2. *PaCrd1* transcript level was analyzed by qPCR and normalized to *PaPorin* expression level. Data represent mean ± SD (wild type: n = 6, *ΔPaIap*: n = 3).

To investigate whether or not longevity of *ΔPaIap* is linked to the observed changes in PL metabolism, we set out to analyze the role of PaIAP in the CDP-DAG pathway. First, we compared the abundance of PaCRD1 in isolated mitochondria of the wild type and the *PaIap* deletion strain and found an approximately 2.5-fold increase of PaCRD1 in the deletion mutant (Figure 5A,B). In contrast, transcript levels did only increase slightly but not significantly (Figure 5C). These data indicate that the accumulation of PaCRD1 does not occur due to enhanced transcription, e.g., triggered by retrograde signaling between mitochondria and the nucleus. The PaCRD1 accumulation rather reflects the mutant’s inability to degrade this protein. We also found elevated amounts of other proteins involved in PL biosynthesis like PaPSD1 and PaMDM35 (Figure S5). This is not surprising as PaIAP homologues are known for proteolytic degradation of a variety of proteins located in both mitochondrial membranes. For instance, *Arabidopsis* FTSH4 was demonstrated to degrade proteins involved in mitochondrial protein import, energy metabolism and membrane organization [75]. Yeast YME1 was specifically shown to be responsible for the turnover of components of mitochondrial lipid metabolism like PL transporter UPS1 and UPS2 [51], as well as PE synthase PSD1 [65] and CL remodeling acyltransferase TAZ1 [76]. Additionally, human YME1L was demonstrated to be enriched especially in mitochondria of cardiac and skeletal muscle tissues [77], supporting its role as proteolytic regulator in active mitochondria.

A closer look at the CL levels in our lipidomics data revealed that in each biological replicate of the wild type, variations in the amount of PaCRD1 had virtually no impact on mitochondrial CL level. However, in *ΔPaIap*, CL levels of the lipidomic analysis behaved proportionally to each replicate’s PaCRD1 level (Figure S6). This implies that in wild-type *P. anserina*, there are multiple stages of PL biosynthesis in which PaIAP can interfere, so that, ultimately, CL levels can be kept steady at approximately 1.5%. However, after deletion of *PaIap* the ability to regulate CL biosynthesis proteolytically is impaired.

In order to further verify the proteolytic regulation of PaCRD1 experimentally, we monitored PaCRD1 abundance in isolated mitochondria of the wild type and the *ΔPaIap* strain 24 h after the inhibition of cytoplasmic protein biosynthesis with cycloheximide (CHX). In wild-type strains, we observed a decrease of mitochondrial PaCRD1 by approximately 30% (Figure S7). Unfortunately, due to the already low amount of PaCRD1 without CHX, results were not significant. However, in *ΔPaIap* CHX treatment had no effect on PaCRD1 levels indicating that the decrease of PaCRD1 in the wild type most likely depends on PaIAP activity.

### 3.4. The Effect of ΔPaIap on Longevity Is Artificially Achieved in Wild-Type Strains Growing on Non-Fermentable Glycerol

**Figure 6:**
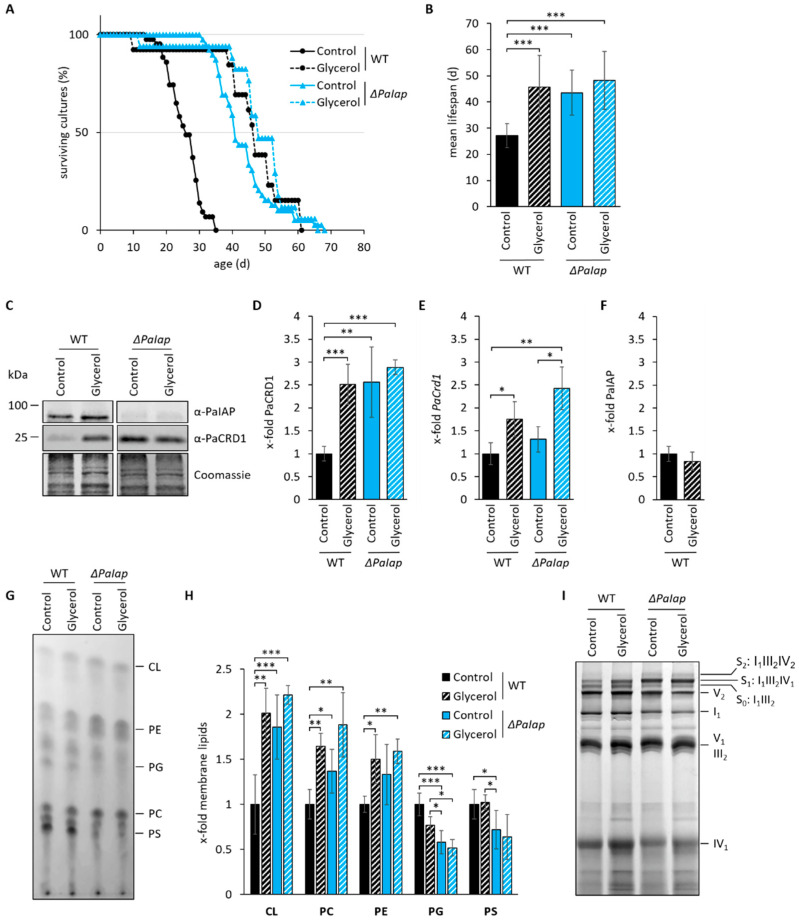
Absence of PaIAP alters mitochondrial protein and PL composition comparable to those observed in respiratory active mitochondria. (**A**): Lifespan analysis of wild type (WT) and *ΔPaIap* grown on M2 either with dextrin (control, glycolytic condition) or glycerol (respiratory condition) as primary carbon source at 27 °C. (**B**): Mean lifespan from (**A**). Data represent mean ± SD (wild type (control): n = 43; wild type (glycerol): n = 13; *ΔPaIap* (control): n = 39; *ΔPaIap* (glycerol): n = 17). (**C**): Western blot analysis of isolated mitochondria of 7-day-old WT and *ΔPaIap* grown on CM medium with either glucose (control) or glycerol as primary carbon source. Coomassie staining was used as loading control. (**D**): Quantification of PaCRD1 in (**C**). PaCRD1 levels were determined and related to wild type (set to 1). Data represent mean ± SD (control: n = 5; glycerol n = 3). (**E**): *PaCrd1* transcript analysis of 5-day-old wild type and *ΔPaIap* grown on M2 medium either with dextrin (control) or glycerol. *PaCrd1* transcript level was analyzed by RT-qPCR and normalized to *PaPorin* expression level. Data represent mean ± SD (wild type (control): n = 6; *ΔPaIap* (control): n = 3; glycerol: n = 3). (**F**): Quantification of PaIAP in (**C**). PaIAP levels were determined and related to wild type (set to 1). Data represent mean ± SD (n = 3). (**G**): One-dimensional TLC analysis of mitochondrial PLs of the wild type (WT) and *ΔPaIap* grown on glucose (control) or glycerol containing CM medium. PL extraction and analysis was performed as described in *Material and Methods*. (**H**): Quantification of PL spots in (**G**). Intensity of each PL spot was measured and related to the intensity of the whole track. Wild-type values of each PL were set to 1. Data represent mean ± SD (wild type (control): n = 5; wild type (glycerol): n = 3; *ΔPaIap* (control): n = 11; *ΔPaIap* (glycerol): n = 4). (**I**): BN-PAGE analysis of isolated mitochondria of 7-day-old WT and *ΔPaIap* strains grown either under glycolytic (Control) or respiratory (Glycerol) conditions. Subscripted numbers represent degree of oligomerization per RC complex. S_0_ to S_2_ represent different RCS depending on amount of complex IV per supercomplex. * *p* < 0.05; ** *p* < 0.01; *** *p* < 0.001.

Next, we investigated whether the observed longevity effect in the *PaIap* deletion mutant is connected to alterations in mitochondrial function. In human muscle cells, a shift from glycolytic to respiratory conditions was previously shown to reprogram cellular energy metabolism favoring oxidative phosphorylation [78], which requires proper mitochondrial function. Thus, we investigated if growth on glycerol as non-fermentable carbon source affects the lifespan of *ΔPaIap*. Interestingly, respiratory growth caused no significant lifespan effects in the deletion mutant (Figure 6A). In contrast, the mean lifespan of wild-type strains was nearly doubled to approximately 46 days (Figure 6B), which resembles the mean lifespan of *ΔPaIap* under both, glycolytic as well as respiratory conditions. Lifespan extension on non-fermentable glycerol was observed earlier [79] and is most likely due to the relief of carbon catabolite repression.

These findings led us to hypothesize that establishing strictly respiratory conditions may mimic the mechanism, on which the longevity of *ΔPaIap* is based. Since deletion of *PaIap* leads to a significant alteration of the mitochondrial PL profile, we examined a possible connection between metabolic state and PL biosynthesis by investigating PaCRD1 levels in wild-type and *ΔPaIap* mitochondria grown either under glycolytic or respiratory conditions. Indeed, we found that growth on non-fermentable glycerol significantly increases the amount of PaCRD1 in the wild type (Figure 6C,D) to a similar degree as in the PaIAP ablated mutant. However, unlike in *ΔPaIap*, this increase of PaCRD1 results from an elevated transcription rate of its nuclear-encoded gene (Figure 6E) and not from a reduction or even complete absence of PaIAP (Figure 6F). This clearly demonstrates that the wild type reacts to the environmental changes by elevating PL biosynthesis at the transcriptional level.

To assess whether this transcriptional activation eventually results in altered mitochondrial PL composition, we performed thin layer chromatography (TLC) analysis of isolated mitochondria of each strain under both, glycolytic and respiratory conditions. We observed an overall increase of the membrane lipids CL, PE and PC in the wild type grown on glycerol (Figure 6G,H), supporting our assumption of transcriptional induction of genes involved in PL synthesis by respiratory growth. This conclusion is in agreement with results from studies in *S. cerevisiae*, which likewise demonstrated elevated levels of these PLs, in strains growing on non-fermentable ethanol, glycerol or lactate [14,80,81]. As expected, PL levels in *ΔPaIap* were only slightly affected, as they were already elevated under glycolytic conditions. Overall, our data support the hypothesis, that the increased lifespan of *ΔPaIap* is strongly related to the enforced enhancement of PL metabolism.

Previous studies revealed an increased formation of RCS in the *ΔPaIap* mutant under glycolytic conditions [10]. Since we hypothesized that the elevated PL levels of *ΔPaIap*, especially CL, induce RCS assembly, we tested whether cultivation under respiratory conditions has similar effects on RCS formation in the wild type. Therefore, we performed BN-PAGE analysis of isolated mitochondria of wild-type and *ΔPaIap* strains grown under both, glycolytic and respiratory conditions. As predicted, the formation of RCS was induced in wild-type strains grown on glycerol, especially of supercomplex S_0_ and S_1_ (Figure 6I). However, proportions of each supercomplex slightly differ between wild type and *ΔPaIap*, since the latter one is mostly enriched in complex IV containing S_1_. In yeast, it was already shown that constitution of complex IV containing RCS is highly dependent on the amount and species of CL in the IMM [82]. Therefore, it is conceivable that the observed enrichment of CL (72:8) in *ΔPaIap* (Figure 4C) is responsible for this specific RCS profile. Additionally, considering complex IV as a potential substrate of PaIAP [83,84] the enrichment of S_1_ may be due to the ablation of the protease and the resulting impairment of complex IV proteolysis.

Since CL represents an important factor for different lipid-protein interactions, we also examined whether increased CL levels alone are sufficient to mimic the phenotype of *ΔPaIap.* Thus, we generated a mutant, which constitutively overexpresses the *PaCrd1* gene (Figure S8A) and performed western blot analysis as well as TLC of isolated mitochondrial fractions. Overexpression of *PaCrd1* resulted in a nearly 10-fold increase of PaCRD1 (Figure S8B,C) and a 2- to 2.5-fold elevation of mitochondrial CL (Figure S8D,E), while PE and PC levels remain unchanged. Surprisingly, *PaCrd1* overexpression did not affect the mean lifespan at all (Figure S8F), indicating that general increase of CL levels is not sufficient to increase lifespan. This leads to the assumption that either this overexpression mutant lacks certain CL species like tetra-linoleoyl CL, which may be required for proper function, or combined elevation of all three PLs is required to lead to the *ΔPaIap*-like phenotype.

Such a dependency would not be surprising as mitochondrial function is known to be affected by each of these PLs. CL and PE for instance, are not only relevant for specific lipid-protein interactions that stabilize components of the oxidative phosphorylation [85,86]. The cone-shaped structure of these non-bilayer PLs also induces negative curvature of membranes, therefore contributing to cristae formation and inner membrane enlargement [87,88,89]. Moreover, they are also required for proper mitochondrial dynamics through membrane fusion and fission [16,90,91]. Elevation of PC synthesis is not only beneficial, but indispensable as well, as it ensures a steady PE/PC ratio, thus counteracting membrane stiffening as well as consequent induction of PL-dependent ER stress [92,93,94].

### 3.5. PaIAP as an Adaptive Regulator Is Necessary for Short-Term Reaction on Changing Environmental Conditions

**Figure 7:**
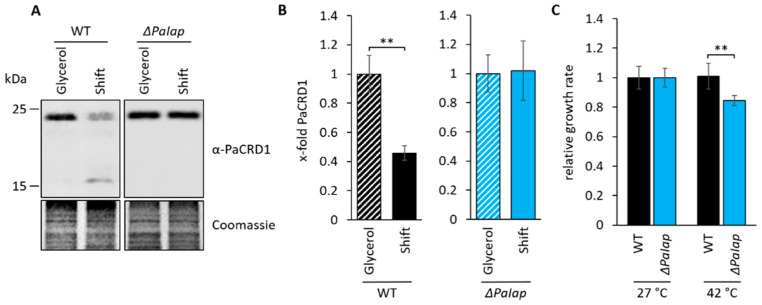
PaIAP is required for short-term adaption to changing environments (**A**): Western blot analysis of isolated mitochondria of 7-day-old wild type (WT) and *ΔPaIap* grown on glycerol and subsequently shifted to glucose as primary carbon source (Shift). Coomassie staining was used as loading control. (**B**): Quantification of PaCRD1 in (**A**). PaCRD1 levels were determined and related to glycerol control (set to 1). Data represent mean ± SD (n = 3). ** *p* < 0.01. (**C**): Heat stress analysis of 9-day-old WT and *ΔPaIap* strains, grown in CM liquid medium were exposed to 27 as well as 42 °C for 5 h and subsequently placed on M2 solid medium for 3-day regeneration at 27 °C. Growth rates of both strains were determined and related to each strain’s control (set to 1). Data represent mean ± SD (n = 4, except WT (42 °C) n = 8). ** *p* < 0.01.

The absence of PaIAP and subsequent elevation of mitochondrial PL synthesis was demonstrated to cause major benefits concerning mitochondrial quality, eventually promoting longevity. These observations led us to ask for a biological role of PaIAP as a major regulator of PL metabolism as well as its evolutionary value. In nature, organisms are generally facing continuous changes of environmental conditions, including fluctuations in temperature or the availability and quality of nutrients. This situation makes it essential that organisms are equipped with appropriate pathways allowing them to efficiently adapt to these fluctuations. Since maintaining proper membrane plasticity requires continuous regulation of PL proportions, we investigated the role of PaIAP for the fungus’ ability to rewire its metabolism according to nutrient availability. Therefore, we cultivated both, the wild type and *ΔPaIap* for 3 days under strictly respiratory conditions (glycerol) to induce PL biosynthesis and subsequently shifted each strain to medium containing glucose as sole carbon source. After 2 days of incubation, we isolated and analyzed mitochondria from the corresponding cultures. We hypothesized that exposing respiring *P. anserina* to glycolytic conditions might induce a metabolic shift favoring glycolytic growth thereby readjusting PL biosynthesis. Indeed, western blot analyses of these isolates reveal that in contrast to *ΔPaIap*, wild-type strains adapted to the altered carbon source by proteolytic degradation of excessive PaCRD1 (Figure 7A,B), which is most likely accompanied by a reduction of CL biosynthesis. These results are in agreement with studies of human YME1L in which this protease was shown to be responsible for degradation of mitochondrial proteins in glycolytically growing pancreatic ductal adenocarcinoma (PDAC) cells [95]. The immediate degradation of PaCRD1 demonstrates that *P. anserina* presumably prefers glycolytic growth in the presence of glucose. This might as well explain why enhanced RCS formation in *ΔPaIap* has no significant impact on ROS release on glucose as primary carbon source. Additionally, it supports our hypothesis of PaIAP being an adaptive regulator for rapid environmental alterations. Still, these observations did not clarify the importance of PaIAP for the organism, especially since the *ΔPaIap* mutant demonstrated healthy growth even after shifting primary carbon sources.

Interestingly, previous studies revealed that the absence of PaIAP vastly reduced the mutant’s heat stress tolerance [10]. Data showed that growth at 37 °C led to a significantly decreased lifespan accompanied by impaired health parameters, such as growth rate and sexual reproduction. Since membrane composition, especially concerning membrane fluidity, plays a key role for adaption to temperature fluctuations, we concluded that the mutant’s temperature-sensitivity might result from the inability to regulate PL metabolism in order to preserve membrane integrity at elevated temperatures. Being homologous to yeast YME1, PaIAP is also suggested to act as an important component of mitochondrial quality control by degradation of denatured or unfolded proteins, which is also essential for heat stress adaptability. To investigate this kind of short-term adaption more closely, we exposed 9-day-old wild-type and *ΔPaIap* strains to 42 °C for 5 h and examined growth rates after 3 days of regeneration. We found that *ΔPaIap* is characterized by a significantly reduced growth rate (approximately 20%) compared to wild-type strains (Figure 7C). Thus, we concluded that deletion of *PaIap* and consequent elevation of PL biosynthesis presumably enhances mitochondrial quality on different levels, which ultimately results in longevity under constant and optimized laboratory conditions. However, this state appears to be hardly reversible due to the absence of PaIAP as key regulator; hence, being fatal in fluctuating natural environments.

### 3.6. Elevated Mitochondrial Turnover Ensures Proper Quality Control despite Absence of PaIAP

**Figure 8:**
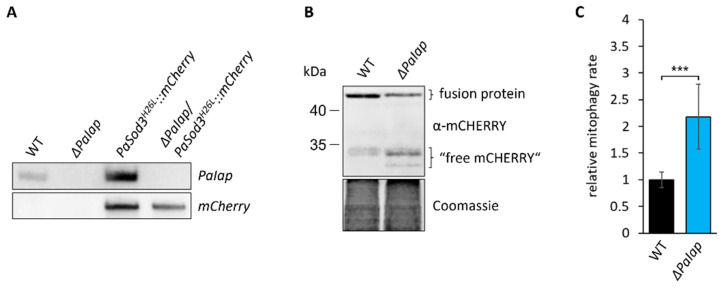
Loss of PaIAP enhances mitochondrial turnover. (**A**): Southern blot analysis of HindIII-digested DNA of the generated mitophagy reporter strain *ΔPaIap/PaSod3^H26L^::mCherry*. (**B**): Western blot analysis of isolated protein extracts of 6-day-old *PaSod3^H26L^::mCherry* (here WT) and *ΔPaIap/PaSod3^H26L^::mCherry* (here *ΔPaIap*) grown in liquid CM medium. Coomassie staining was used as loading control. (**C**): Quantification of mitophagy rate in (**B**). Mitophagy rate was determined by ratio of “free mCHERRY” to total “free mCHERRY” + PaSOD3^H26L^::mCHERRY (fusion protein). Data represent mean ± SD (*PaSod3^H26L^::mCherry* n = 9; *ΔPaIap/PaSod3^H26L^::mCherry* n = 10). *** *p* < 0.001.

Finally, we were confronted with the question on which functions of PaIAP make a difference to its yeast homologue YME1, especially, since *Yme1* deletion in yeast was shown to cause a strongly reduced chronological and replicative lifespan [96,97]. However, effects on PL metabolism and temperature sensitivity are similar to our observations in *P. anserina* [98,99]. In yeast, a key function of YME1 is ATG32 mediated induction of mitophagy. YME1 is responsible for processing the mitochondrial mitophagy receptor ATG32, which is located in the OMM and interacts with ATG11, a scaffold protein for selective autophagy [100]. In yeast cells lacking YME1, unprocessed ATG32 was shown to accumulate, accompanied by a significant reduction of mitochondrial turnover. Mitophagy is an essential component for cellular quality control as it ensures degradation of dysfunctional mitochondria ensuring proper energy metabolism. In *P. anserina*, mild induction of mitophagy was also shown to be part of a hormetic stress response, which contributes to a delayed aging process [28]. Since no ATG32 homologues are known in filamentous fungi like *P. anserina*, we hypothesized that mitophagy induction might still be functional in *ΔPaIap*, acting as a “backup” mechanism compensating for the loss of PaIAP activity. We addressed this possibility and performed a “mCHERRY cleavage assay” to monitor mitophagy rate in young wild type and deletion mutants producing a mCHERRY-tagged inactive variant of the mitochondrial superoxide dismutase PaSOD3 (Figure 8A). In these strains, vacuolar turnover of mitochondria results in the degradation of the PaSOD3^H26L^::mCHERRY fusion protein. During this process, the mCHERRY moiety remains stable and can be quantified as a measure for mitophagic flux. We found that not only is *ΔPaIap* able to induce mitophagy, but the ratio of mitochondrial turnover was also more than doubled compared to the wild type (Figure 8B,C). Although, previous studies revealed that longevity of *ΔPaIap* is not solely dependent on autophagy [101], our findings suggest that autophagic turnover of damaged mitochondria is able to compensate the deficit in proteolytic mitochondrial quality control of *ΔPaIap*, circumventing an accumulation of dysfunctional mitochondria, as it is shown in yeast *ΔYme1* mutants. This is supported by western blot analysis of total protein extracts, which revealed neither increase nor reduction of mitochondrial proteins in *ΔPaIap* compared to the wild type (Figure S9). Thus, studying the *ΔPaIap* mutant allows us to establish a direct correlation between PL metabolism and aging in eukaryotic organisms.

## 4. Conclusions

In this study, we investigated the impact of genetically altered PL homeostasis on organismic aging, thereby establishing a link between mitochondrial PL levels and lifespan of the fungal aging model *P. anserina*. In this process, we introduced the mitochondrial i-AAA protease PaIAP as an adaptive short-term regulator, being essential for proteolytic adjustment of PL biosynthesis to energetic requirements and natural temperature fluctuations. However, we propose that, under certain environmental conditions, enhanced biosynthesis of CL, PE, and PC through the absence of PaIAP causes a shift in mitochondrial physiology, ultimately leading to an extended healthspan.

## Data Availability

The data presented in this study are available upon reasonable request from the corresponding author.

## Supplementary Materials

The following are available online at https://www.mdpi.com/article/10.3390/cells10102775/s1, Figure S1. Verification of a *PaTaz1* deletion strain; Figure S2. Loss of PaCRD1 causes accumulation of PaGEP4; Figure S3. Loss of PaIAP does not affect ROS release from mycelia; Figure S4. Elevated CL levels result in improved iron tolerance; Figure S5. Impact of PaIAP ablation on level of components of PL metabolism; Figure S6. PaIAP proteolytically regulates CL biosynthesis; Figure S7. PaIAP is required to degrade PaCRD1; Figure S8. Enhanced CL synthesis has no impact on lifespan of *P. anserina*; Figure S9. Deletion of *PaIap* does not alter relative amount of different mitochondrial proteins (all figures summarized in “Figure S1–S9.docx”); Table S1. Mitochondrial lipid profiles; Table S2. CL species and double bonds; Table S3. Additional PL species and double bonds (all tables summarized in “Table S1–3.xlsx”).
