# Supplementary material for: Mitochondrial Phospholipid Homeostasis Is Regulated by the i-AAA Protease PaIAP and Affects Organismic Aging
Source: Cells. 2021 Oct 16;10(10):2775. doi: 10.3390/cells10102775 (PMC8534651; doi:10.3390/cells10102775)
Supplement: Supplementary file 1 [file cells-10-02775-s001.zip › Figures S1-S9_Revised#2.pdf]

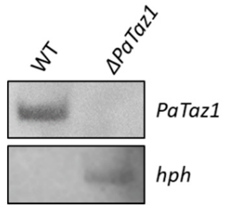

Figure S1. Verification of a *PaTaz1* deletion strain. Southern blot analysis with HindIII-digested DNA of *P. anserina* wild-type (WT) and *ΔPaTaz1* strains demonstrating replacement of the *PaTaz1* ORF with a hygromycin resistance gene (*hph*) in the deletion mutant.

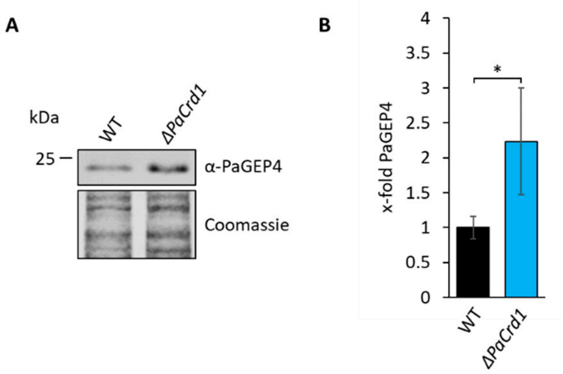

Figure S2. Loss of PaCRD1 causes accumulation of PaGEP4. **A:** Western blot analysis of isolated mitochondria of 7-day-old wild type and *ΔPaCrd1*. Coomassie staining was used as loading control. **B:** Quantification of PaGEP4 (UniProt B2B6T3) in **A**. PaGEP4 levels were determined and related to wild type (set to 1). Data represents mean ± SD (n = 3). *p < 0.05.

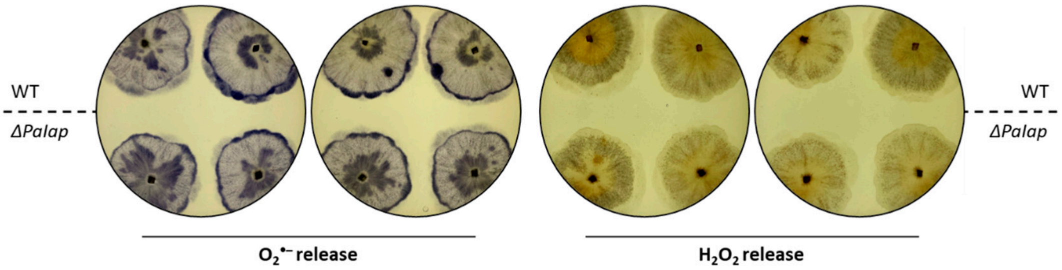

Figure S3. Loss of PaIAP does not affect ROS release from mycelia. Visualization of released superoxide anion free radical ($O_2^{\bullet-}$) and hydrogen peroxide (H_2O_2) in juvenile wild type (WT) and *ΔPaIap* by histochemical NBT and DAB staining (each n = 4).

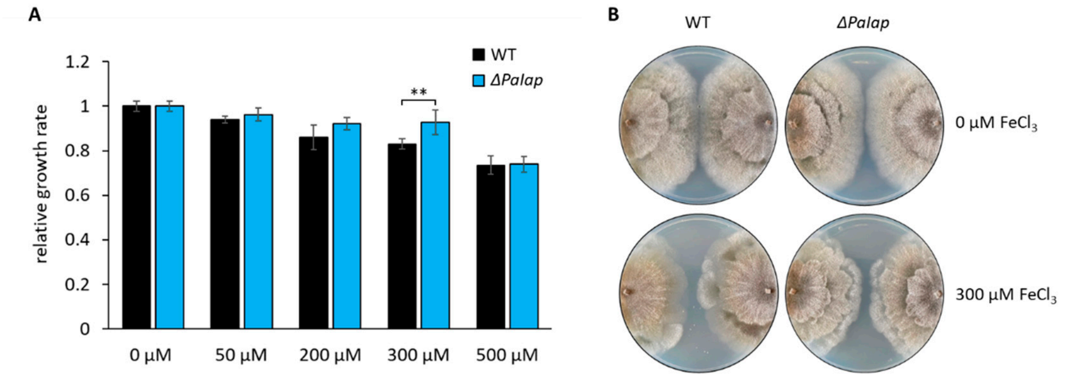

Figure S4. Elevated CL levels result in improved iron tolerance. **A:** Freshly germinated wild type (WT) and *ΔPaIap* strains, were grown on M2 medium supplemented with 0-500 µM $FeCl_3$ at 27 °C under constant light. After 3 d, growth rates of both strains were determined and related to each strain's growth rate on 0 µM $FeCl_3$ (set to 1). Data represents mean ± SD (n = 6). **p < 0.01. **B:** Phenotypes of WT and *ΔPaIap* strains grown on M2 medium with 0 or 300 µM $FeCl_3$ after 7 d.

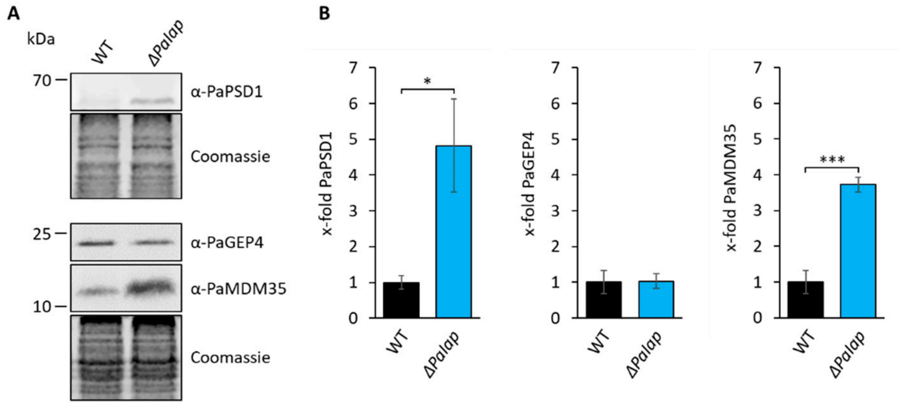

Figure S5. Impact of PaIAP ablation on level of components of PL metabolism. **A:** Western blot analysis of isolated mitochondria of 7-day-old wild-type and *ΔPaIap* strains. Coomassie staining was used as loading control. **B:** Quantification of PaPSD1 (UniProt B2B7S1), PaGEP4 and PaMDM35 (UniProt A0A090CMV1) in **A**. Protein levels were determined and related to wild type (set to 1). Data represents mean ± SD (n = 3). *p < 0.05, ***p < 0.001.

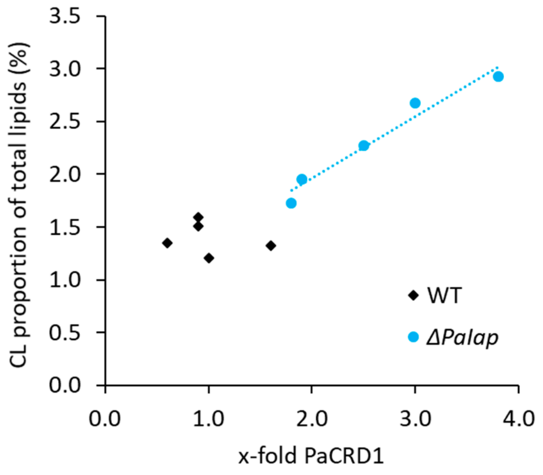

Figure S6. PaIAP proteolytically regulates CL biosynthesis. In *ΔPaIap* a clear correlation between relative PaCRD1 level (see Fig. 5) (average of wild type set to 1) and relative CL proportion can be observed, which is not present in wild type (WT). Both values were obtained from each strain used for lipidomic analysis.

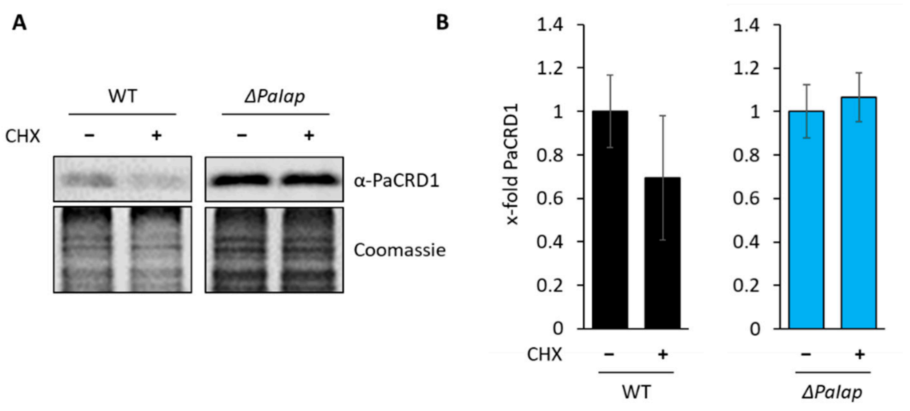

Figure S7. PaIAP is required to degrade PaCRD1. **A:** Western blot analysis of 7-day-old wild type and *ΔPaIap* mitochondria without (-) and 24 h after cycloheximide (CHX) treatment (+). Coomassie staining was used as loading control. **B:** Quantification of PaCRD1 in **A**. PaCRD1 levels were determined and related to control without CHX (set to 1). Data represents mean ± SD (n = 3).

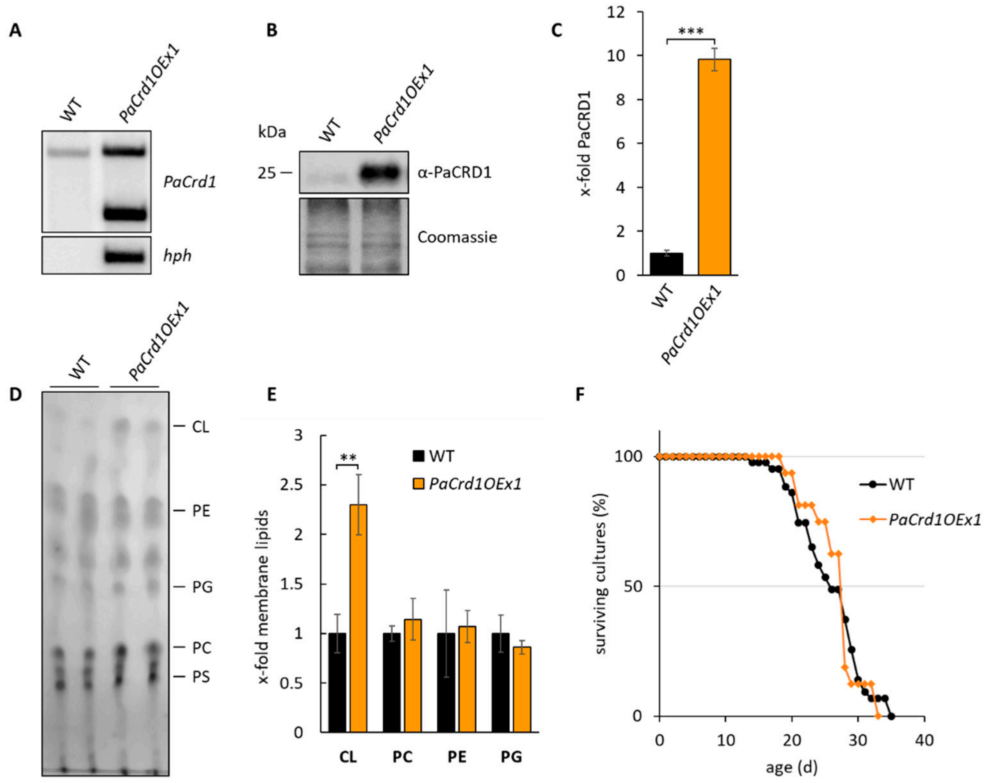

Figure S8. Enhanced CL synthesis has no impact on lifespan of *P. anserina*. **A:** Southern Blot analysis with HindIII-digested DNA of wild type (WT) and *PaCrd1OEx1* shows ectopic integration of an additional copy of *PaCrd1* in *PaCrd1OEx1* compared to the wild type. **B:** Western blot analysis of isolated mitochondria of 7-day-old wild type and *PaCrd1OEx1*. Coomassie staining was used as loading control. **C:** Quantification of PaCRD1 in **B**. PaCRD1 levels were determined and related to wild type (set to 1). Data represents mean ± SD (n = 3). ***p < 0.001. **D-E:** One dimensional TLC analysis of mitochondrial PLs of WT and *PaCrd1OEx1* grown on glucose containing CM medium. PL extraction and analysis was performed as described in *Material and Methods*. Intensity of each PL spot was measured and related to the intensity of the whole track. WT values of each PL were set to 1. Data represents mean ± SD (n = 3). **p < 0.01. **F:** Lifespan analysis of WT and *PaCrd1OEx1* grown on M2 at 27 °C under constant light.

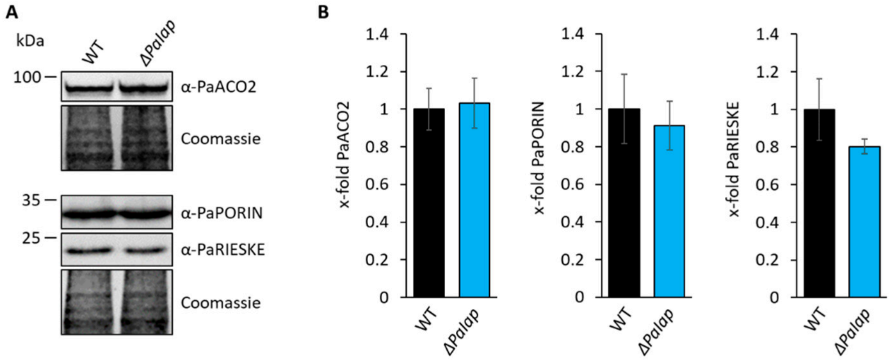

Figure S9. Deletion of *PaIap* does not alter relative amount of different mitochondrial proteins. **A:** Western blot analysis of total protein extracts of 6-day-old wild type (WT) and *ΔPaIap* grown in liquid CM medium. Coomassie staining was used as loading control. **C:** Quantification of mitophagy rate in **B**: Quantification of PaACO2 (UniProt B2VLF5), PaPORIN (UniProt B2B736) and PaRIESKE (UniProt B2B3E7) in **A**. Protein levels were determined and related to wild type (set to 1). Data represents mean ± SD (n = 4).
